# Supplementary material for: Combining Clinical and Genetic Data to Predict Response to Fingolimod Treatment in Relapsing Remitting Multiple Sclerosis Patients: A Precision Medicine Approach
Source: J Pers Med. 2023 Jan 6;13(1):122. doi: 10.3390/jpm13010122 (PMC9861774; doi:10.3390/jpm13010122)
Supplement: Supplementary file 1 [file jpm-13-00122-s001.zip › jpm-2065221-Table S1.pdf]

Supplementary Table S1: **Genetic signature prioritized by the machine learning analysis**

rs10915271
rs549148
rs720887
rs2488429
rs203849
rs2075992
rs4653996
rs853469
rs12038267
rs10802244
rs2028380
rs1561322
rs2307466
rs17035884
rs4971790
rs1045920
rs2234500
rs2708943
rs6740091
rs3755158
rs6705679
rs10460317
rs263767
rs11886926
rs13081043
rs9845152
rs8180087
rs333327
rs17509456
rs13314733
rs2079169
rs13115793
rs10000695
rs2004794
rs2124935
rs11957276
rs268791
rs918520
rs262753
rs9261578
rs1264318
rs3129296
rs7761731

rs9474433
rs10944770
rs9361875
rs2787897
rs2235261
rs2076193
rs9456931
rs683369
rs760812
rs12699798
rs6963278
rs10226192
rs2237279
rs12538253
rs587499
rs11136431
rs4512398
rs969365
rs7017723
rs4149256
rs4998
rs4236939
rs1487173
rs12678366
rs1009759
rs2033543
rs11793821
rs10780660
rs4625093
rs3765101
rs12770204
rs8463
rs10906326
rs2244677
rs8177062
rs11203042
rs11192823
rs17104648
rs2237997
rs3741097
rs10161275
rs10848775
rs10842971
rs12317270

rs11170177
rs4762615
rs4761051
rs1564647
rs2479758
rs9566276
rs2806900
rs943601
rs9318893
rs319561
rs938886
rs17182592
rs8003820
rs12895930
rs11850195
rs1722791
rs17815875
rs2414680
rs4886414
rs4984343
rs8048501
rs187680
rs254353
rs1060253
rs868874
rs9962319
rs4372773
rs11084943
rs501537
rs6511422
rs6055919
rs4809688
rs157640
rs2426778
rs8139582
rs9617449

The table shows the list of the 123 SNPs prioritized by the genetic analysis.
